# A cost-effective oligo-based barcode system for chromosome identification in longan and lychee

**DOI:** 10.1093/hr/uhae278

**Published:** 2024-09-28

**Authors:** Zehuai Yu, Yiying Qi, Yuxuan Wei, Gui Zhuang, Yihan Li, Baiyu Wang, Sehrish Akbar, Yi Xu, Xiuting Hua, Qiutao Xu, Zuhu Deng, Jisen Zhang, Yongji Huang, Fan Yu, Jiannan Zhou

**Affiliations:** Guangxi Key Laboratory for Sugarcane Biology, Guangxi University, State Key Laboratory for Conservation and Utilization of Subtropical Agro-Bioresources, 100 University East Road, Nanning 530004, China; College of Agriculture, Fujian Agriculture and Forestry University, No.15 Shangxiadian Road, Cangshan District, Fuzhou 350002, China; Guangxi Key Laboratory for Sugarcane Biology, Guangxi University, State Key Laboratory for Conservation and Utilization of Subtropical Agro-Bioresources, 100 University East Road, Nanning 530004, China; Guangxi Key Laboratory for Sugarcane Biology, Guangxi University, State Key Laboratory for Conservation and Utilization of Subtropical Agro-Bioresources, 100 University East Road, Nanning 530004, China; Guangxi Key Laboratory for Sugarcane Biology, Guangxi University, State Key Laboratory for Conservation and Utilization of Subtropical Agro-Bioresources, 100 University East Road, Nanning 530004, China; Guangxi Key Laboratory for Sugarcane Biology, Guangxi University, State Key Laboratory for Conservation and Utilization of Subtropical Agro-Bioresources, 100 University East Road, Nanning 530004, China; Guangxi Key Laboratory for Sugarcane Biology, Guangxi University, State Key Laboratory for Conservation and Utilization of Subtropical Agro-Bioresources, 100 University East Road, Nanning 530004, China; Guangxi Key Laboratory for Sugarcane Biology, Guangxi University, State Key Laboratory for Conservation and Utilization of Subtropical Agro-Bioresources, 100 University East Road, Nanning 530004, China; Guangxi Key Laboratory for Sugarcane Biology, Guangxi University, State Key Laboratory for Conservation and Utilization of Subtropical Agro-Bioresources, 100 University East Road, Nanning 530004, China; Guangxi Key Laboratory for Sugarcane Biology, Guangxi University, State Key Laboratory for Conservation and Utilization of Subtropical Agro-Bioresources, 100 University East Road, Nanning 530004, China; Guangxi Key Laboratory for Sugarcane Biology, Guangxi University, State Key Laboratory for Conservation and Utilization of Subtropical Agro-Bioresources, 100 University East Road, Nanning 530004, China; Guangxi Key Laboratory for Sugarcane Biology, Guangxi University, State Key Laboratory for Conservation and Utilization of Subtropical Agro-Bioresources, 100 University East Road, Nanning 530004, China; Ministerial and Provincial Joint Innovation Centre for Safety Production of Cross-Strait Crops, College of Geography and Oceanography, Minjiang University, Minhou District, Fuzhou 350108, China; Guangxi Key Laboratory for Sugarcane Biology, Guangxi University, State Key Laboratory for Conservation and Utilization of Subtropical Agro-Bioresources, 100 University East Road, Nanning 530004, China; Key Laboratory of Tropical Fruit Biology (Ministry of Agriculture), South Subtropical Crops Research Institute, Chinese Academy of Tropical Agricultural Sciences, Mazhang District, Zhanjiang 524091, China

## Abstract

Oligonucleotide (Oligo)-based fluorescence *in situ* hybridization (FISH) represents a highly effective methodology for identifying plant chromosomes. Longan is a commercially significant fruit species, yet lacking basic chromosomal markers has hindered its cytogenetic research. In this study, we developed a cost-effective oligo-based system for distinguishing chromosomes of longan (*Dimocarpus longan* Lour., 2*n* = 2*x* = 30). For this system, each synthesized oligo contained two chromosome-specific sequences that spanned a distance of over 200 kb, and a PCR-based flexible amplification method coupled with nested primers was used for probe labeling. The use of these oligo-based barcodes enabled the marking of 36 chromosomal regions, which allowed for the unambiguous distinction of all 15 chromosomes in both longan and lychee (*Litchi chinensis* Sonn., 2*n* = 2*x* = 30) species. Based on the identification of individual chromosomes, we constructed karyotypes and detected genome assembly errors involving the 35S ribosomal RNA gene (35S rDNA) in longan and lychee. Developing oligo-based barcodes offers considerable promise for advancing cytogenetic research in longan, lychee, and their related species. Furthermore, this cost-effective synthesis system can be referred to the development of new oligo libraries among other species.

## Introduction

Performing karyotyping on chromosomes from nonhomologous groups is crucial for cytological research, as it has been used to demonstrate the evolutionary changes within a species and its related species. In plants, determining chromosomal karyotypes involves identifying individual chromosomes using chromosome banding or fluorescence *in situ* hybridization (FISH) [1]. Compared to chromosomal banding, DNA probes in FISH have shown greater efficacy in elucidating crucial cytogenetic information in plants. Genomic DNA, repetitive sequences, and bacterial artificial chromosome (BAC) libraries have traditionally been the primary probes used for chromosome identification using FISH [2–4]. Genomic DNA probes are often used to differentiate between chromosomes in various lineages; however, they are incapable of detecting individual chromosomes. Despite the widespread use of repetitive sequences and BAC clones for chromosomal identification, their use across species has been hindered by their drawbacks, including the production of unstable signals. Repetitive sequences often result in varied signals in closely related species, and the signal patterns of BAC clones may create annoying interference [1]. As a result, these probes cannot generate a consistent signal pattern across all closely related species.

**Figure 1:**
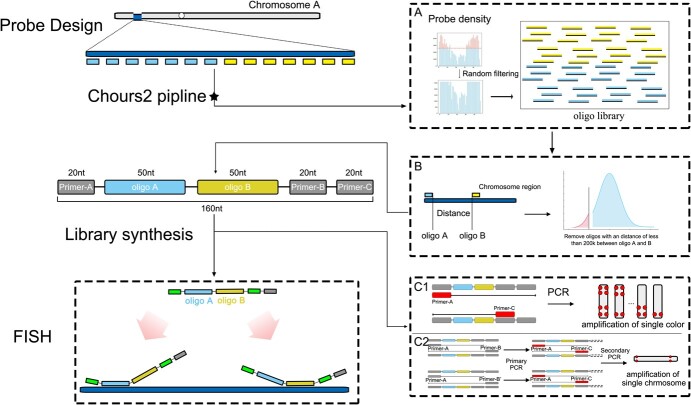
Cost-effective oligo system design schematic. Each synthetic oligo consists of two oligos (oligo-A and oligo-B), one forward primer (Primer-A), and two reverse primers (Primer-B and Primer-C). (A) Oligo library filtering process. The density of the oligo library is 2.5 oligos per kb. (B) Schematic selection of oligo A and B. Synthetic oligos with intervals shorter than 200 kb are omitted for FISH performance. (C) Scheme of primer introducing and PCR amplification. Primer-A and Primer-C are used to amplify the whole pool of different colors/modification probe oligos (C1). Primer-A and Primer-B are used to amplify one individual chromosome or segment (C2). Primer-B and Primer-B′ correspond to distinct sections of the primer, respectively.

The advancements in bioinformatics and genomics have led to the development of synthetic oligo technology, which offers a reliable FISH probe for identifying chromosomes in sequenced genome species [5]. Oligo-based probes, produced from genome sequences and consisting of single-copy or low-copy sequences, have reduced signal noise compared to conventional FISH probes. These probes have been increasingly used in plant genetics and genomics research across various plants [6–12]. In addition, oligo-based probes have shown effective in distantly related species that have undergone divergence of 5–8 million years (Mya) or even 12 Mya [6].

Longan (*Dimocarpus longan Lour.*) and lychee (*Litchi chinensis Sonn.*) are significant tropical fruits in southern China and Southeast Asia [13]. Referred to as “treasured health fruits” due to their exceptional fruit appearance, aroma, and taste, these fruits have a cultivation history in China dating back over a millennium [13]. Both lychee and longan, belonging to the *Sapindaceae* family, share a close genetic relationship and exhibit similar phenotypes. They are commonly found in similar ecological environments. However, the lack of cytogenetic markers hinders studying relationships between lychee, longan, and other related fruits within the *Sapindaceae* family. Consequently, establishing a standard karyotype analysis for longan and its relatives based on chromosome identification has not been feasible.

This study developed a cost-effective methodology that employs oligonucleotides extracted from the longan genome to generate distinctive hybridization patterns on discrete chromosomes using FISH. The methodology entailed pairing each 50 nt oligonucleotide sequence to create a single synthetic oligonucleotide of 100 nt. Through multiplex PCR of the oligonucleotides, 36 chromosome-specific region signals were successfully generated, facilitating the identification of all 15 chromosomes in longan and lychee. Furthermore, the research revealed the precise localization of the 35S ribosomal RNA gene (35S rDNA) on individual chromosomes, identifying an error in genome assembly related to 35S rDNA sites in longan and lychee. This cost-effective oligo-based barcode system markedly reduced costs by half compared to previous oligo pool synthesis, providing a valuable resource for cytogenetic investigations in diverse plant species.

## Results

### Development of oligo-based barcode probes in longan

**Figure 2:**
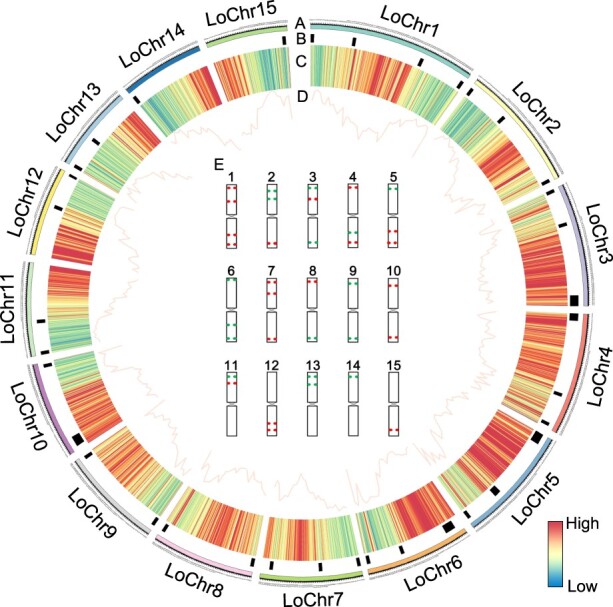
Characteristics of barcode oligos in the longan genome. The outermost track (A) delineates the 15 pseudo-chromosomes of longan, scaled in megabases (Mb). The innermost tracks (B) highlight the selection of oligo barcode sites. A heatmap (C) represents the distribution of oligo counts, while a line plot (D) illustrates the density of these oligos across the genome. (E) features ideograms that demonstrate the design of the oligo barcode probe, with colors indicating signals for subsequent FISH analysis. The oligo count and density are determined within 1 Mb windows for a comprehensive analysis.

To create probes specific to individual chromosomes, a total of 1 023 364 oligonucleotides (50 nucleotides in length) were identified across all 15 chromosomes of the longan genome using the Chorus2 pipeline [5, 14] (Fig. 1). The longan genome was divided into 1 Mb segments to remove duplicate oligos, ensuring each segment contained a maximum of 2500 oligos per chromosome-specific region (Fig. 1a). This resulted in a density of 0.98–2.5 oligos per kb within each barcode region (Fig. 2 and Table S1). Then, 50 272 red and 41 278 green probes were pre-selected to identify longan chromosomes (Table S5). Ultimately, a total of 36 chromosomal regions spanning all 15 chromosomes were chosen, allowing the identification of these chromosomes through one FISH experiment.

### Synthesis of oligo-based barcode probes from the cost-effective oligo synthesis system

To reduce the expense of synthesizing the oligo pool, we merged each pair of chosen oligonucleotide sequences (50 nucleotides (nt) in length) into a single sequence (100 nt in length) (Fig. 1B). These combined probes are pooled into one single FISH experiment. In this economical oligo pool, we ensured a minimum separation of 200 kb between DNA sequences on the specified chromosome to prevent competition for hybridization between the neighboring oligos (oligo-A and oligo-B) (Fig. 1B; Fig. S1). Three distinct primers were added to tag the probes and to identify specific chromosomal regions and individual chromosomes within the longan genome (Fig. 1C): (a) Primer-A and Primer-B were employed to amplify the red and green probe oligo pools, respectively. The labeled 36 probes were utilized for recognizing all 15 chromosomes of longan through once FISH; (b) Primer-A and Primer-C were employed to amplify the unique chromosomal signals of chromosomes 1–15. Barcode oligo-FISH probes have been demonstrated to be effective in identifying nonhomologous chromosomes. Each unit of the synthetic pool was composed of 100 nt DNA sequences derived from the longan genome in conjunction with three amplification primers (Table S2). Consequently, an oligo pool comprising 91 550 oligos was constructed and tagged with three distinct primers for chromosome identification.

**Figure 3:**
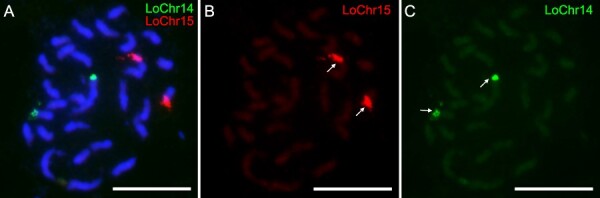
FISH mapping of chromosome-specific probes in longan. (A) Chromosome 14 is visualized with the LoChr14 probe, and chromosome 15 with the LoChr15 probe . (B) Digital separation of the LoChr15 signals from the composite image in A, highlighting chromosome 15. (C) Digital separation of the LoChr14 signals from the composite image in A, highlighting chromosome 14. Bar = 10 μm.

To assess the performance of a cost-effective oligo synthesis system for FISH, we utilized the fluorescently labeled primer pairs to amplify chromosome-specific probes for longan chromosomes 14 and 15, designated as LoChr14 and LoChr15, respectively (Fig. 3; Table S2). These probes were fluorescently labeled with Cy3 (red) for LoChr14 and FAM (green) for LoChr15. Notably, the application of these probes resulted in distinct signals on the metaphase chromosomes of longan, as depicted in Fig. 3. Consistent with our hypothesis, LoChr14 and LoChr15 probes each yielded two pairs of signals on their respective chromosomes (Fig. 3). This outcome indicates the potential of our synthesized oligos for chromosome identification in longan. Subsequently, we employed 2 sets of forward primers (Primer-A) and 21 sets of reverse primers (Primer-C) to amplify all target regions, as listed in Table S2. Each probe generated a clear and specific signal on the corresponding chromosome, corroborating the designed hybridization pattern (Fig. S2).

### Comparative karyotype analysis using oligo-based barcode probes in longan, lychee, and rambutan

**Figure 4:**
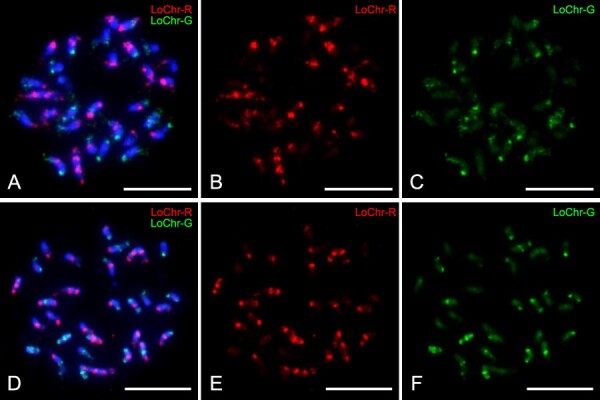
FISH of chromosome-specific oligo barcode probes in longan and lychee species. (A–C) In longan cultivar Shixia, the results of the FISH mapping of chromosome-specific oligo barcode probes are displayed on a mitotic metaphase cell. The original composite image (A) is followed by digitally separated fluorescence signals forthe LoChr-R probe (B) and the LoChr-G probe (C). (D–F) A comparable FISH mapping is presented on a mitotic metaphase cell in the case of the cultivar Ziniangxi lychee. The composite image (D) is accompanied by digitally separated fluorescence signals for the LoChr-R probe (E) and theLoChr-G probe (F). Bar = 10 μm.

**Figure 5:**
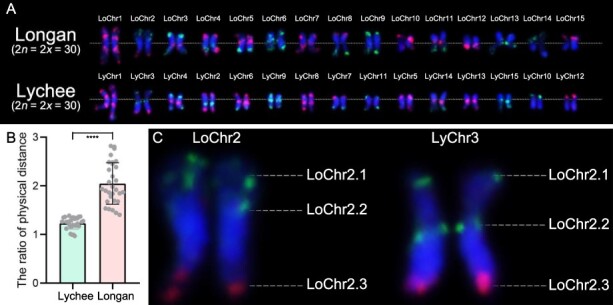
Oligo-based comparative karyotype analysis of longan and lychee. (A) The FISH labeled chromosomes, as originally presented in Fig. 4, have been digitally extracted for detailed analysis. (B, C) This panel presents a comparison of the physical distance ratios between chromosome segments LoChr2.1–LoChr2.2 and LoChr2.2–LoChr2.3 in both longan and lychee. The statistical significance was determined using two-tailed *t*-tests without adjustment for multiple comparisons, with the following notation for *P* values: ^**^P < 0.01, ^***^P < 0.005, and ^****^P < 0.001.

This study explored the karyotypic variations among longan cultivar Shixia, the lychee cultivar Ziniangxi, and the rambutan (*Nephelium lappaceum* L.) cultivar Baoyan7. Utilizing specific primers, we amplified red and green oligo probes designed to discern all 15 chromosomes, aligning with the predefined patterns (Fig. 2; Table S2). FISH with these probes resulted in 36 distinct signals on metaphase chromosomes longan cultivar Shixia, correlating with the expected positions on the 15 pseudomolecules (Fig. 4A–C). Subsequently, we applied oligo-based barcode probes, originally derived from longan, to lychee metaphase chromosomes. The probes generated clearly defined signals and the 15 pseudomolecule chromosomes exhibited similar patterns to that observed in longan (Fig. 5A). Notably, the spatial distribution of the LoChr2 probe segments in longan (the physical distance from LoChr2.1 to LoChr2.2/the physical distance from LoChr2.2 to LoChr2.3) displayed a significantly lower ratio (*P* < 0.001, Student’s *t*-test) compared to lychee (Fig. 5B). This finding suggests that lychee chromosome 3 might have experienced a segmental inversion or a significant expansion of transposable elements (TEs), diverging from the longan karyotype (Fig. 5C).

Subsequently, the relative lengths of chromosomes were quantified, and correlation coefficients were assessed for chromosome comparisons between longan and lychee. The correlation coefficients exhibited considerable variation, ranging from −0.81 to 0.56. In particular, specific probes for chromosomes LoChr2, LoChr3, LoChr5, LoChr10, LoChr13, and LoChr14 demonstrated negative correlations, indicating a divergence in chromosome structure between the two species (Table 1). It is noteworthy that the LoChr2 probe exhibited the most pronounced evolutionary divergence. Furthermore, a comparative analysis of TE characteristics within putative homologous groups of both species was conducted (Fig. S3A and S3B). This analysis revealed a modest positive correlation (*R* = 0.14) between the relative chromosomal lengths and the distribution patterns of TEs across the two species (Fig. S3C).

**Table 1:** Mitotic metaphase chromosome lengths and arm ratios in longan and lychee

Probe^a^	Longan (2*n* = 2*x* = 30)			Lychee (2*n* = 2*x* = 30)			Coefficient of association
	Chromosome^b^	Relative length^d^ (%)	Arm ratio^c^	Chromosome^b^	Relative length^d^ (%)	Arm ratio^c^	
LoChr1	LoChr1	5.78 ± 0.31	0.76 ± 0.15	LyChr1	5.7 ± 0.32	0.89 ± 0.11	0.56
LoChr2	LoChr2	3.75 ± 0.4	0.86 ± 0.08	LyChr3	4.48 ± 0.16	0.79 ± 0.14	−0.81
LoChr3	LoChr3	3.62 ± 0.23	0.88 ± 0.1	LyChr4	3.62 ± 0.21	0.84 ± 0.1	−0.19
LoChr4	LoChr4	3.48 ± 0.23	0.77 ± 0.3	LyChr2	3.64 ± 0.18	0.88 ± 0.06	0.46
LoChr5	LoChr5	3.12 ± 0.33	0.88 ± 0.1	LyChr6	3.07 ± 0.21	0.83 ± 0.1	−0.28
LoChr6	LoChr6	3.08 ± 0.36	0.82 ± 0.09	LyChr9	2.9 ± 0.24	0.82 ± 0.1	0.45
LoChr7	LoChr7	3.52 ± 0.19	0.73 ± 0.28	LyChr8	3.34 ± 0.39	0.86 ± 0.12	0.49
LoChr8	LoChr8	3.82 ± 0.09	0.77 ± 0.19	LyChr7	2.78 ± 0.12	0.87 ± 0.1	0.02
LoChr9	LoChr9	3.4 ± 0.3	0.8 ± 0.16	LyChr11	2.68 ± 0.16	0.88 ± 0.06	0.52
LoChr10	LoChr10	2.48 ± 0.2	0.82 ± 0.15	LyChr5	2.69 ± 0.22	0.81 ± 0.09	−0.68
LoChr11	LoChr11	3.04 ± 0.27	0.8 ± 0.12	LyChr14	3.59 ± 0.31	0.82 ± 0.15	0.62
LoChr12	LoChr12	2.58 ± 0.17	0.76 ± 0.1	LyChr13	2.94 ± 0.27	0.87 ± 0.09	0.30
LoChr13	LoChr13	3.1 ± 0.42	0.74 ± 0.2	LyChr15	2.93 ± 0.18	0.86 ± 0.14	−0.53
LoChr14	LoChr14	2.89 ± 0.23	0.73 ± 0.14	LyChr10	2.81 ± 0.11	0.86 ± 0.09	−0.46
LoChr15	LoChr15	2.57 ± 0.23	0.86 ± 0.1	LyChr12	3.09 ± 0.19	0.8 ± 0.12	0.02

a Numbers 1–15 correspond to the signal patterns in Fig. 2E.

b Chromosomal naming follows the respective references of the genome assemblies.

c Arm ratio is determined by the length of the long arm divided by the length of the short arm for each chromosome.

d Relative length is calculated as 100× (length of each chromosome/total length of all chromosomes).

Furthermore, our examination of metaphase cells from rambutan revealed an absence of detectable signals using the oligo-based barcode probes (Fig. S4). This absence of signal may be attributed to the significant evolutionary divergence of rambutan compared to longan and lychee. This suggests that the probes designed based on longan’s genome may not be sufficiently conserved to hybridize effectively with rambutan chromosomes.

### Reconciling discrepancies between molecular cytogenetics and bioinformatics to reconstruct phylogenetic relationships

**Figure 6:**
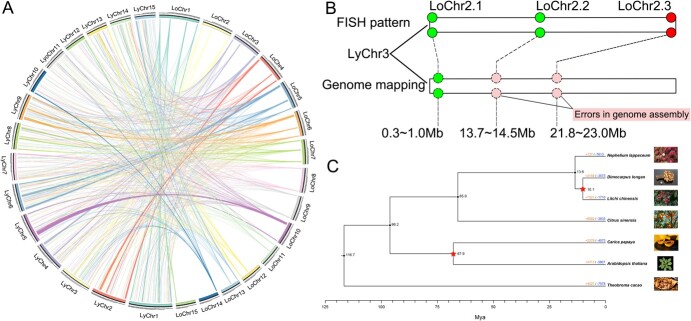
Oligo sequence analysis and longan genome evolution. (A) Longan-specific oligo barcode probe alignments to lychee genome. The circos plot depicts the monoploid genome size in Mb, with chromosomes indexed from the origin at 0 Mb. Chromosomes are differentiated by distinct colors, each representing a unique chromosome group. (B) Comparative chromosomal localization of LoChr2 probe by FISH and genome mapping. (C) The phylogenetic tree presented here elucidates the relationships and approximate divergence times among longan, lychee, and rambutan, providing insights into their evolutionary history.

To corroborate the cytogenetic barcode pattern findings, we conducted a comparative genomic analysis by mapping longan oligo DNA sequences onto the genomes of lychee and rambutan (Fig. 6A; Table S4). Of the 91 550 oligos DNA sequences, 37 211 (40.65%) successfully aligned with the lychee genome, while 1842 sequences (4.95%) were found on non-orthologous chromosomes (Fig. 6A; Table S5). Notably, the longan chromosome segments LoChr2.1, LoChr2.2, and LoChr2.3 each corresponded to positions 0.3–1.0 Mb, 13.7–14.5 Mb, and 21.8–23.0 Mb on LyChr3 of the lychee genome, respectively. (Fig. 6A and B). However, the FISH analysis indicated that LoChr2.2 and LoChr2.3 segments are located at the centromere and the telocentromeric regions, respectively (Figs 5C and 6B), which suggests a potential discrepancy that may be attributed to errors in genome assembly. Furthermore, the relatively low mapping rate to the rambutan genome (27.13%, corresponding to 24 836 sequences) generated no distinct FISH signals, indicating either a more distant phylogenetic relationship or challenges in sequence alignment (Tables S4 and S5).

Phylogenetic trees are indispensable for accurately estimating divergence times and delineating relationships among plant species. Despite prior investigations into the divergence times between longan and lychee and lychee and rambutan, a clear understanding of the relationships and divergence times among these three species has yet to be established [15]. In this study, we constructed a phylogenetic tree utilizing single-copy orthologous protein sequences. Our findings indicate that the divergence of longan and lychee from rambutan occurred at ~13.6 Mya, with a subsequent divergence between longan and lychee occurring at ~10.1 Mya (Fig. 6C). These findings suggest that oligo-based barcode probes derived from longan may be a viable approach for chromosome identification in species that have diverged within the last 10.1 million years. However, their utility may be constrained in species that are more distantly related, particularly beyond a divergence threshold of 13.6 Mya.

### Genome-wide comparative FISH mapping of 35S rDNA in longan and lychee

**Figure 7:**
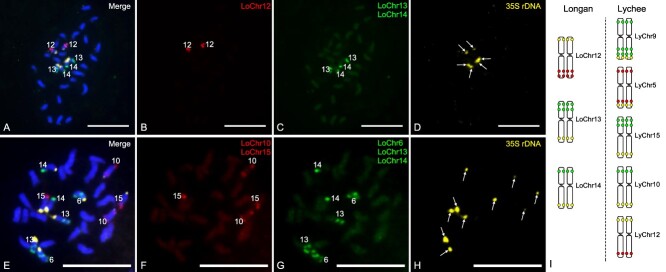
Comparative FISH detection of 35S rDNA in longan and lychee genomes. (A–D) In longan, FISH was performed on metaphase chromosomes using chromosome-specific probes for LoChr12 , LoChr13, and LoChr14, along with the 35S rDNA probe, which appears yellow due to the overlap of signals. (E–H) In lychee, FISH was similarly conducted with chromosome-specific probes for LoChr10, LoChr15, and LoChr6, with additional probes for LoChr13 and LoChr14, and the 35S rDNA probe. Note that the LoChr13 and LoChr14 probes are labeled in green, indicating the same color but potentially different chromosome regions. (I) The schematic represents the chromosomal locations of the 35S rDNA sites in both longan and lychee, highlighting the differences in their distribution. Bar = 10 μm.

The 35S rDNA has been demonstrated to be a reliable molecular marker for evaluating plant ploidy and the characterization of interspecific relationships [16]. We aimed to investigate the distribution of 35S rDNA sites in the genomes of longan and lychee. To achieve this objective, we conducted FISH analysis using 35S rDNA probes in conjunction with longan-specific oligo probes in metaphase chromosomes of longan (Fig. 7A–D) and lychee (Fig. 7E–H). The examination yielded 6 distinct 35S rDNA loci in longan (Fig. 7D) and 10 in lychee (Fig. 7H). Through co-location analysis, it was determined that in longan, these loci were found on chromosomes 12, 13, and 14, while in lychee, they were distributed across chromosomes 5, 9, 10, 12, and 15 (Fig. 7I).

**Figure 8:**
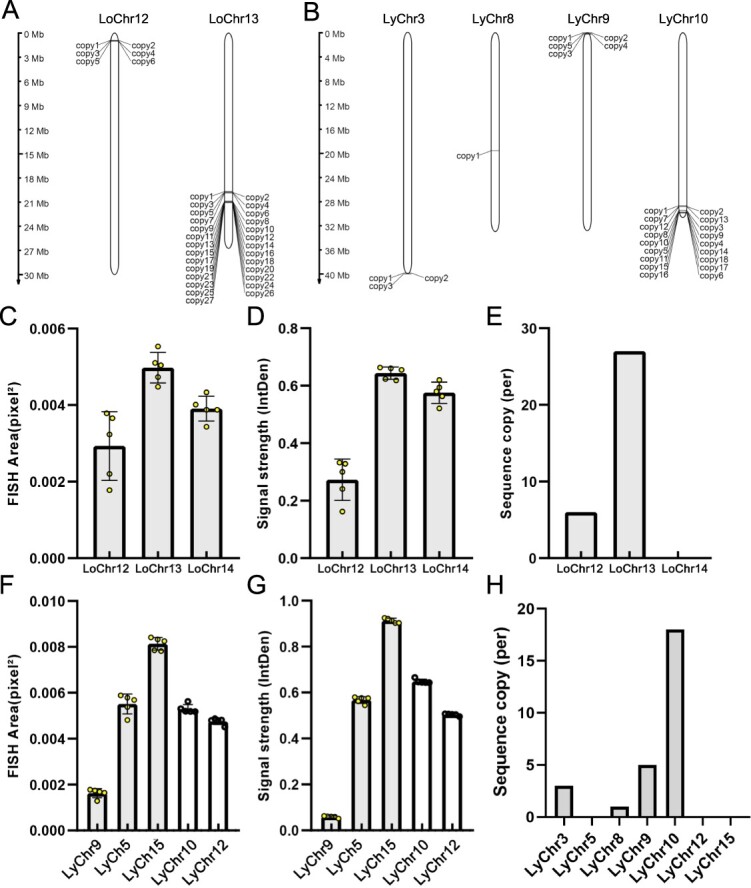
Comparative analysis of 35S rDNA loci using FISH and genome assembly data. (A) FISH mapping of 35S rDNA sequences on the longan genome, illustrating their chromosomal distribution. (B) FISH mapping of 35S rDNA sequences on the lychee genome shows a distinct chromosomal localization pattern. In the longan genome, comparisons of (C) FISH signal sizes and (D) signal strengths among longan chromosomes. (E) Quantifying 35S rDNA copies in longan chromosomes highlights the variation in copy number across different chromosomes. In the lychee genome, a comparison of (F) FISH signal sizes and (G) signal strengths among lychee chromosomes. (H) Quantification of 35S rDNA copies in lychee chromosomes, emphasizing the distribution and copy number variation on the chromosomes.

We performed a comparative investigation of 35S rDNA loci using FISH and genome assembly data. A total of 33 35S rDNA copies were detected in the longan genome. Specifically, six copies were located on the terminal short arm of chromosome 12, while 27 copies were found on the terminal long arm of chromosome 13 (Fig. 8A and E). This finding indicates that the longan genome assembly lacks 35S rDNA sites on chromosome 14, as shown in Fig. 7I. A total of 27 copies of 35S rDNA were found within the lychee genome. These copies were located on the short arms of chromosomes 3 and 9 and chromosomes 8 and 10. The largest number of 18 copies was found on chromosome 10 (Fig. 8B and H). However, the lychee genome assembly did not include the 35S rDNA loci on chromosomes 5, 10, and 12 (Fig. 7I). In addition, we determined the physical dimensions of the 35S rDNA clusters by evaluating the strength and extent of the FISH signals. Longan chromosomes were ranked in terms of physical size as LoChr13 > LoChr14 > LoChr12. Similarly, lychee chromosomes were ranked as LyChr15 > LyChr10 > LyChr5 > LyChr12 > LyChr9. These rankings represent the relative abundance and distribution of 35S rDNA copies (Fig. 8C, D, F, and G).

## Discussion

FISH has emerged as the predominant approach for chromosome identification in plants [17]. The careful selection of FISH probes holds significant importance in cytogenetic research. At present, oligo-based probes derived from sequenced genomes have demonstrated their value as a powerful tool in cytogenetic research across various plant species. Oligonucleotides that target multi-specific chromosomal regions (referred to as “barcodes”) or entire chromosomes (termed “painting”) are commonly employed for chromosome identification. Nevertheless, the financial burden associated with synthesizing oligo pools may present a significant obstacle to using oligo-based probes, particularly in species with a high fundamental chromosome number. For example, a set of 27 000 oligonucleotides could cost approximately $1500 [9, 17]. Therefore, it is crucial to optimize the capacity of the pool. In this study, we have developed a method for synthesizing oligo pools that combines two DNA sequences (50 nt each) into a single oligo (100 nt in length) (Fig. 1). This novel approach allows us to reduce the cost of synthetic oligo pools by half.

Typically, there are two techniques available for labeling oligo probes. The T7 *in vitro* transcription labeling method generates a single-stranded oligo probe that is tagged and has a high level of specificity. Nevertheless, the process of labeling single-stranded probes required several sequential procedures and the use of various experiment kits, hence increasing its complexity and cost. The second method involves PCR amplification, which generates double-stranded probes that greatly improve FISH signals and decrease costs [17, 18]. In this investigation, we utilized Primer-A and Primer-B to amplify the barcode probes, and Primer-A and Primer-C to amplify particular chromosome-specific probes. This approach allowed us to avoid the supererogatory expense of labeling the probes based on PCR amplification. Using multiplex PCR amplification probes, we successfully identified all 15 chromosomes (Fig. 4) and differentiated between individual chromosomes (Fig. 3).

Identification of karyotype differences is conducive to a better understanding of the evolution among species. Traditional FISH probes, such as BAC clones and repetitive sequences, have been an impossible task for self and closely related species. Oligo-based FISH probes have recently been developed for massive plant chromosome research. Chromosome painting probes have revealed fission and evolution in the *Saccharum spontaneum*, in which the basic chromosome number reduced from 10 to 8 [6, 8, 19]. Using bulked oligo probes, several chromosome rearrangements have been identified among *Vigna*, Legume, Triticeae, and other species [9, 20, 21]. In this study, we developed a cost-effective oligo synthesis system for chromosome identification in longan and lychee, enabling the detection of karyotype differences between the two species during their chromosomal evolution (Fig. 5). By incorporating divergence times inferred from the phylogenetic tree (Fig. 6C), we demonstrated that these probes can effectively distinguish closely related species that diverged within 10.1 Mya.

The accuracy and integrity of assembled repetitive sequences are of paramount importance in the evaluation of genome assembly quality [22, 23]. Despite technological advancements that have enabled telomere-to-telomere (T2T) genome assemblies in certain plant species, a significant number of genomes remain lacking in precise assembly [24]. Molecular cytogenetics is a valuable tool for identifying errors in genome assembly [17]. This technique employs specific probes to examine genome structure, including species ploidy and potential chromosome recombination, with the objective of facilitating genome assembly [19, 25, 26]. Moreover, FISH can facilitate the mapping of unanchored scaffolds or contigs [27]. For example, molecular cytogenetics has been successfully employed to unravel the ultra-complex sugarcane genome [19, 23, 26]. Xin *et al*. [27] utilized oligo-FISH to map the entirety of unanchored sequences associated with the poplar sex chromosome. In this study, a combination of cytogenetic and genomic methods was employed to identify assembly errors in lychee chromosome 3 (Figs 6B and 7I). In the longan genome, FISH analysis demonstrated that the 35S rDNA sequences were present on chromosomes 12, 13, and 14 but absent on chromosome 14 in the genome assembly (Figs 7I and 8A). In the lychee genome, chromosomes 9 and 10 were concurrently identified through cytological and bioinformatic analyses as being associated with 35S rDNA. The highest number of 35S rDNA sequences was identified on chromosome 10 of the lychee plant (Fig. 8H), which was inconsistent with the results of cytological observations. Moreover, the 35S rDNA sequences in the lychee genome were not detected on chromosomes 5, 12, and 15, suggesting that the 35 rDNA sequences were not covered by the genome assembly.

## Materials and methods

### Plant materials and chromosome preparation

Longan accession ShiXia (2*n* = 2*x* = 30), lychee accession Ziniangxi (2*n* = 2*x* = 30), and rambutan accession Baoyan01 (2*n* = 2*x* = 32) were utilized in the current investigation. The specimens were upheld within the Longan germplasm resources collection center situated at the South Subtropical Crops Research Institute of CATAS (Chinese Academy of Tropical Agricultural Science) in Zhanjiang, Guangdong Province, China. Root tips originating from areas of active cell division were gathered and subjected to a saturated solution of paradichlorobenzene and α-bromonaphthalene at room temperature (RT), around 25°C for 2 hours and 30 minutes, and subsequently fixed in Carnoy’s fixative solution (alcohol: acetic acid = 3:1). Mitotic chromosome slides were prepared following the established methodology [6].

### Development and synthesis pipeline for oligo library

We selected the special 45-nt oligos using Chorus2 software (add ref or website link) based on the reference genome of Longan “HHZ” cultivar with specific parameters (−-homology 75, −-step 5, −-length 50) [5, 14]. To eliminate potentially repetitive sequences, we applied the ChorusNGSfilter.py and ChorusNGSselect.py scripts to the Illumina short-read sequencing data of the lychee “Feizixiao” cultivar (~66X coverage) and the longan “JDB” cultivar (~50X coverage) using stringent filters (−q 0.1, −p 0.9, −d 25) [28, 29]. In addition, we excised excess oligos exceeding a density threshold of 2.5 per kilobase using a sliding window approach with a window size of 1 MB and a step of 1 MB. We then determined the genomic distance between oligo A and oligo B, retaining only those pairs with a distance of 200 kb or more to construct our oligo library. Each chromosomal region was distinguished by unique primers, which are detailed in Tables S2 and S3. The oligo library was expertly synthesized by CustomArray Inc. (GenScript Corp, Nanjing, China), and the barcode probe was visualized using Python (https://www.python.org/) and Circos (https://circos.ca/software/download/circos/).

### Probe preparation and FISH

The specific cost-effective oligo probes were amplified using a PCR protocol that has been previously described [18]. For FISH, the oligo probes were labeled with the direct label Cy3 and FAM dyes. The 35S rDNA plasmids of sugarcane (*Saccharum officinarum* L.) were labeled with Cy3-dUTP [6] via the nick translation method. The FISH procedure was adapted from a previously established protocol [17]. The mitotic chromosome slide was denatured at 65°C for 90 seconds in the initial round of hybridization, and the denaturation time was reduced to 30 seconds in the subsequent round. The hybridization mixture comprised 70% formamide, 10% 20× SSC (pH 7.0), 20% dextran sulfate, and 100 ng of each probe, with a total volume of 10 μL applied per slide. Subsequently, the mixture was preheated to 90°C for 7 minutes and then placed on ice. Subsequently, the mitotic chromosomes on the slides were air-dried and counterstained with DAPI in VectaShield antifade solution (Vector Laboratories, USA). The FISH signals were captured with a COSMO™ CMOS camera (Teledyne Princeton Instruments, USA) using an Olympus BX53 LED microscope (Olympus Corporation, USA). Subsequently, the images were processed using Adobe Photoshop CS (Adobe Systems Incorporated, USA).

### Oligo mapping and alignment analysis

The barcode sets of oligo sequences were aligned and compared among the genomes of *D. longan*, *L. lychee*, and *N. lappaceum* using the Burrow-Wheeler Aligner (BWA) (https://github.com/lh3/bwa) with the mem pipeline. The genome was then divided into 100-kb windows, and the alignments were visualized using Circos software version 0.69–8 (https://github.com/vigsterkr/circos).

### Phylogenetic and gene family analysis

A phylogenetic tree was constructed for seven species: *N. lappaceum*, *D. longan*, *L. chinensis*, *Citrus sinensis*, *Carica papaya*, *Arabidopsis thaliana*, and *Theobroma cacao*. The single-copy orthologous genes were identified by Orthofinder [30] with the parament “-S diamond”. Then, RAxML [31] was applied to construct ML tree with the “msa” model. A total of 1000 bootstrap replicates were applied. This ML tree was used as an input to r8s [32], to compute the divergent time. The divergence times for *A. thaliana* and *C. papaya* (67.9 Mya) and *D. longan* and *L. chinensis* (10.1 Mya) were employed as calibration points. CAFÉ [33] was employed to identify gene families that have undergone expansions or contractions among the seven plant genomes with the paraments: -p 0.05 -t 4 -l. The amino acid and CDS sequences for these species were obtained from the NCBI database at https://ftp.ncbi.nlm.nih.gov/genomes/refseq/plant/.

### TE annotation

A combination of evidence-based search and *ab initio* prediction approaches was employed to identify TEs in the longan and lychee genomes. In the evidence-based search, both genomes were compared against the Repbase database (Repbase - GIRI(girinst.org)) using the RepeatMasker software (version 4.0.5) (RepeatMasker Home Page). To conduct ab initio prediction, a consensus sequence library was constructed using RepeatModeler (open-1.0.8) (RepeatModeler Download Page (repeatmasker.org)) with the parameter “-engine ncbi”.

### Chromosomal relative length and correlation coefficient

The relative chromosome length and arm ratio were determined using established methodologies [27]. Ten completed metaphase cells from each species were utilized to measure the relative chromosome length and arm ratio between longan and lychee. Chromosome measurements were obtained using the software ImageJ (ImageJ). The correlation coefficients were calculated using the Pearson correlation coefficient in the SciPy.stats.pearsonr() function, as detailed in the SciPy v1.14.0 manual (pearsonr — SciPyv1.14.0 Manual).

### Sequence structure and phylogenetic relationship analysis of 35S rDNA

To identify the 35S rDNA units in the genomes of *D. longan* and *L. chinensis*, we employed a BLASTN-based genomic screening approach utilizing the following sequences as query templates:

I. The 18S rDNA sequence from rice (GenBank Accession: X00755.1);

II. The 25S rDNA sequence from rice (GenBank Accession: M11585.1);

III. The ITS1–5.8S-ITS2 sequence from *Brachypodium distachyon* (GenBank Accession: JN187608.1);

IV. The intergenic spacer (IGS) sequence from *B. distachyon* (GenBank Accession: KX263276) [34].

The sequences were then assembled into a composite structure reflecting the 18S-ITS1–5.8S-ITS2-25S-IGS rDNA unit. BLASTN (Nucleotide BLAST: Search nucleotide databases using anucleotide query (nih.gov)) was employed to identify potential 35S rDNA units within the genomes of *D. longan* and *L. chinensis*. The process entailed aligning the known rDNA structural sequences against the genomic sequences of the two species, utilizing these as a reference. Any hits with a coverage value below 0.8 were disregarded to ensure accuracy. The number of 18S rDNA sequence hits was deemed to be indicative of the 35S rDNA unit’s copy number.

## Supplementary Material

Web_Material_uhae278

## Data Availability

All relevant data are included in the article and its supporting materials.
