# Supplementary material for: A cost-effective oligo-based barcode system for chromosome identification in longan and lychee
Source: Hortic Res. 2024 Sep 28;12(1):uhae278. doi: 10.1093/hr/uhae278 (PMC11750958; doi:10.1093/hr/uhae278)
Supplement: Web_Material_uhae278 [file web_material_uhae278.zip › Supplementary_file.docx]

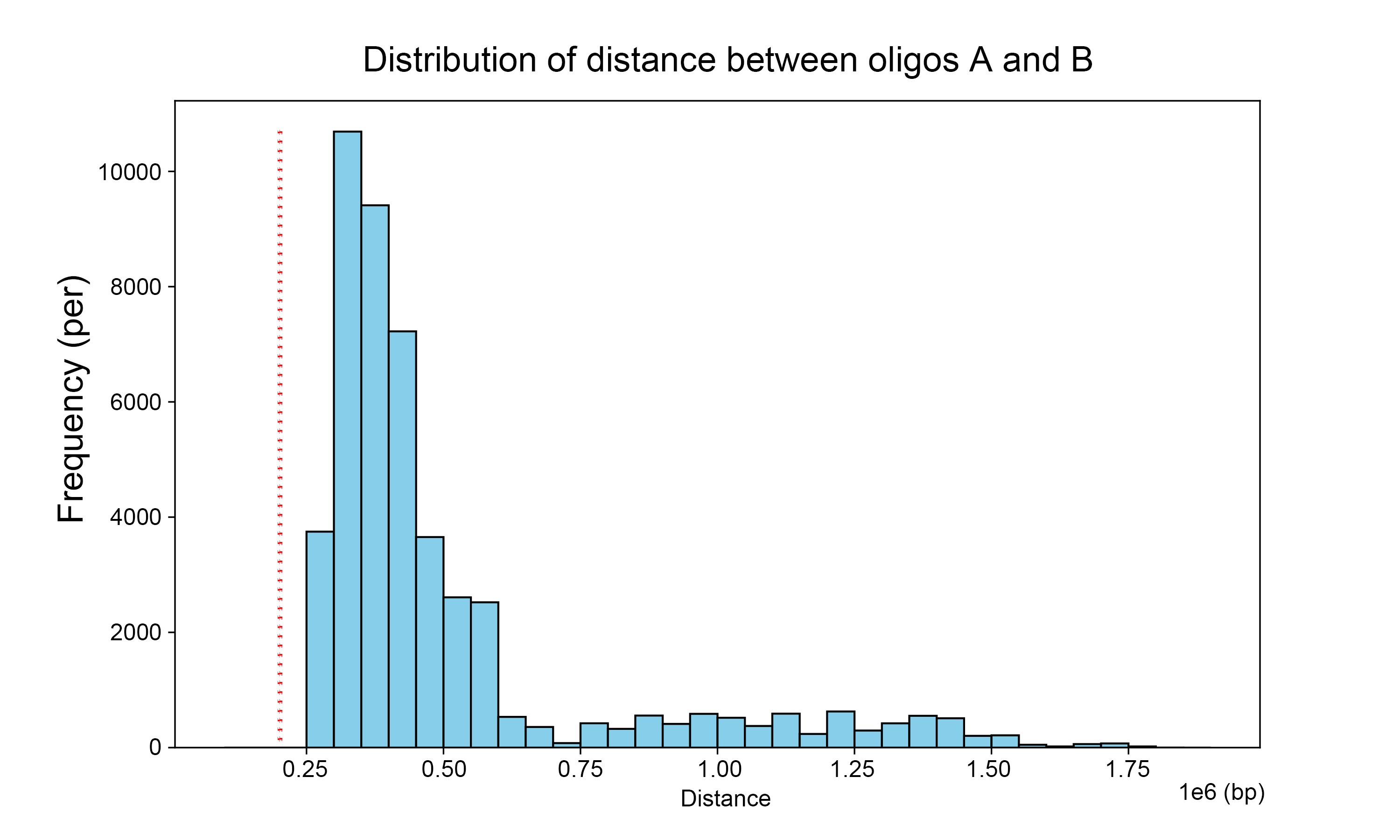


**Figure S1 The frequency of distance between oligos A and B in the cost-effective synthesis system.**

**
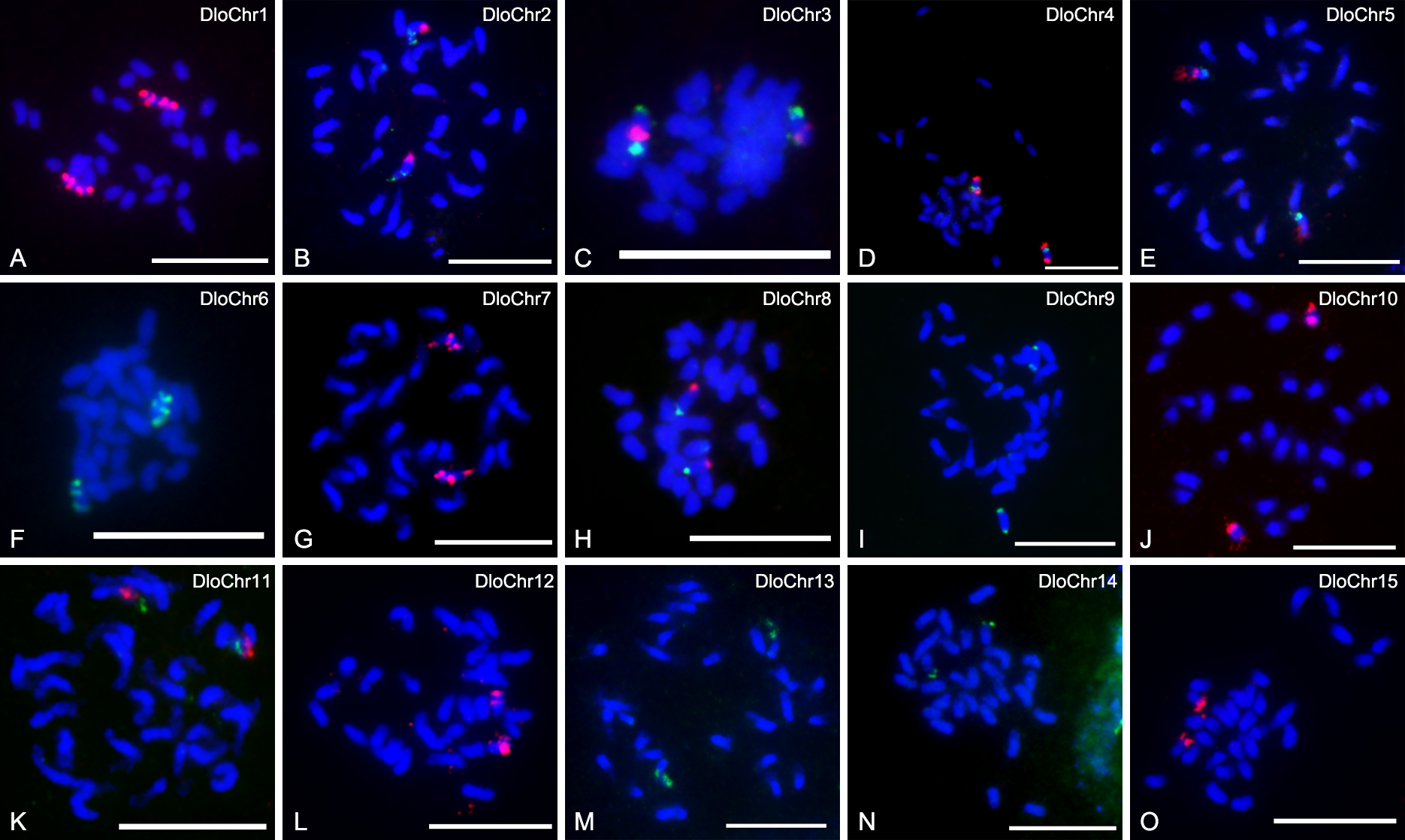
**

**Figure S2 FISH to identify all 15 chromosomes in the longan metaphase cell by special longan oligo barcode probe.**

**
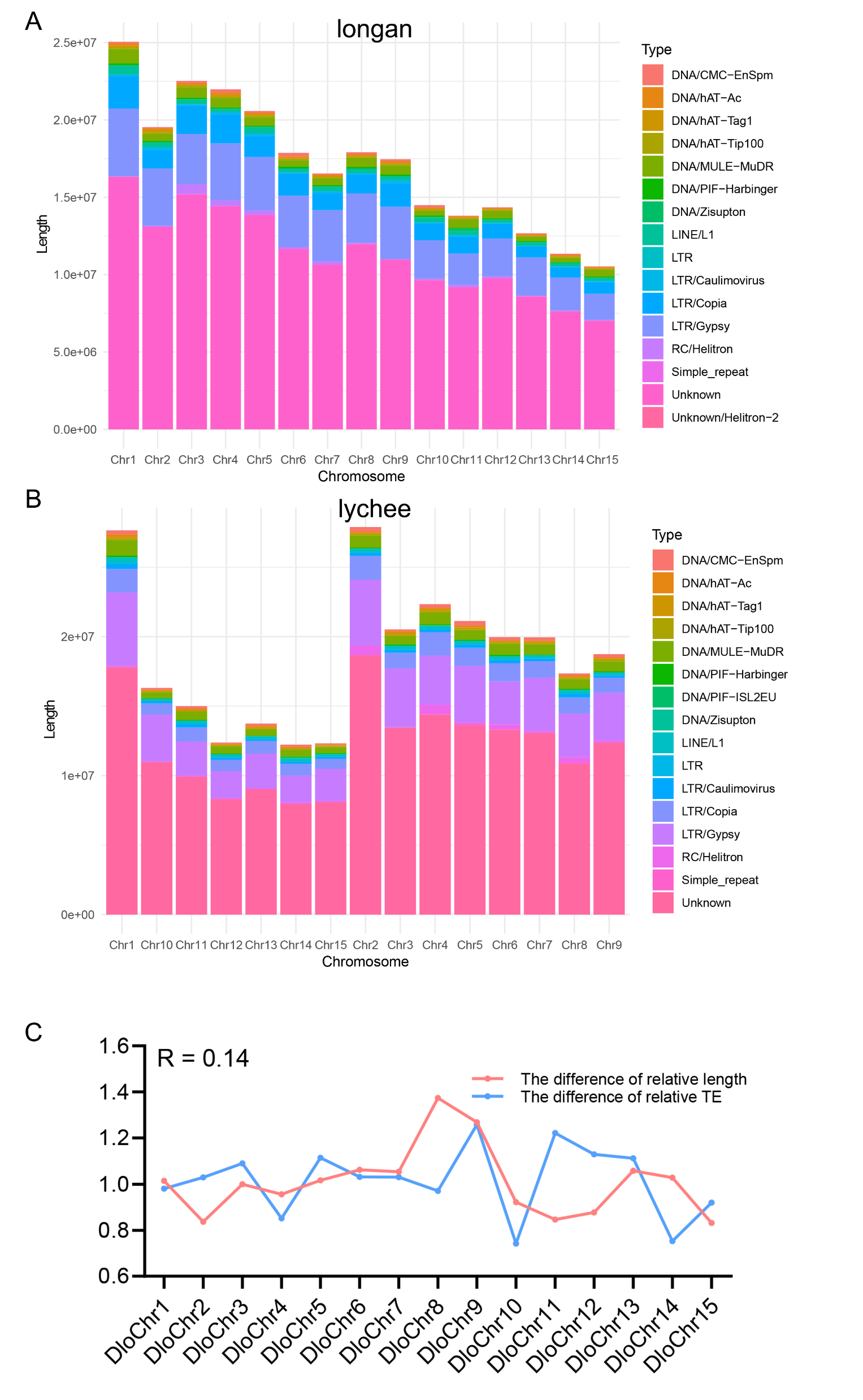
**

**Figure S3 TE expansions linked by physical chromosome length between longan and lychee. Statictics of different TEs along chromosomes in longan (A) and litchi (B). (C) Correlation analysis between chromosome length and TE content.**

**
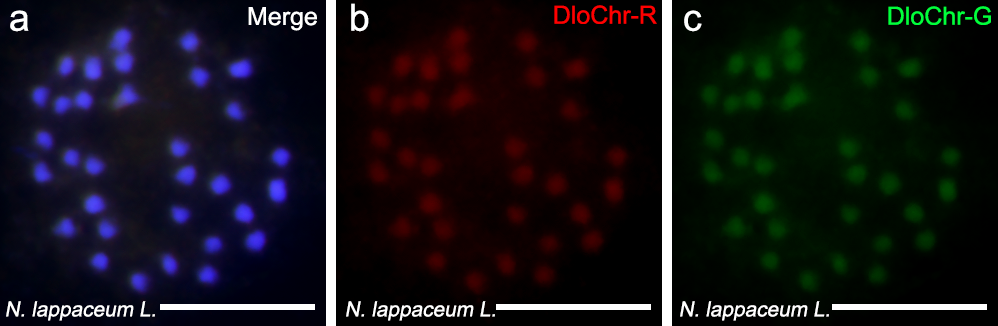
**

**Figure S4 FISH of longan special oligo barcode probe in rambutan.**

**Table S1. Numbers of oligos and chromosome positions of 36** **windows selected for oligo-FISH**

| Probe | Chromosome^a^ | Start (bp) | End (bp) | Number of oligos (per) | Average density^b^ (per/kb) |
| --- | --- | --- | --- | --- | --- |
| LoChr1.1 | LoChr1 | 0 | 1000000 | 2500 | 2.50 |
| LoChr1.2 | LoChr1 | 12000000 | 13000000 | 2500 | 2.50 |
| LoChr1.3 | LoChr1 | 32000000 | 33000000 | 2500 | 2.50 |
| LoChr1.4 | LoChr1 | 45000000 | 46000000 | 2500 | 2.50 |
| LoChr2.3 | LoChr2 | 34000000 | 35000000 | 2500 | 2.50 |
| LoChr3.2 | LoChr3 | 10000000 | 11000000 | 2500 | 2.50 |
| LoChr4.1 | LoChr4 | 0 | 2000000 | 2519 | 1.26 |
| LoChr4.3 | LoChr4 | 32000000 | 33000000 | 2500 | 2.50 |
| LoChr5.2 | LoChr5 | 20000000 | 22000000 | 2500 | 1.25 |
| LoChr5.3 | LoChr5 | 31000000 | 32000000 | 2500 | 2.50 |
| LoChr7.1 | LoChr7 | 0 | 1000000 | 2500 | 2.50 |
| LoChr7.2 | LoChr7 | 11000000 | 12000000 | 2500 | 2.50 |
| LoChr7.3 | LoChr7 | 25000000 | 26000000 | 2500 | 2.50 |
| LoChr8.1 | LoChr8 | 0 | 1000000 | 2500 | 2.50 |
| LoChr10.1 | LoChr10 | 1000000 | 4000000 | 2952 | 0.98 |
| LoChr10.2 | LoChr10 | 25000000 | 26000000 | 2400 | 2.40 |
| LoChr12.1 | LoChr12 | 14000000 | 15000000 | 2500 | 2.50 |
| LoChr12.2 | LoChr12 | 24000000 | 25000000 | 2400 | 2.40 |
| LoChr11.2 | LoChr11 | 9000000 | 10000000 | 2500 | 2.50 |
| LoChr15 | LoChr15 | 21000000 | 22000000 | 2500 | 2.50 |
| LoChr2.1 | LoChr2 | 0 | 1000000 | 2500 | 2.50 |
| LoChr2.2 | LoChr2 | 12000000 | 13000000 | 2420 | 2.42 |
| LoChr3.1 | LoChr3 | 0 | 1000000 | 2500 | 2.50 |
| LoChr3.3 | LoChr3 | 31000000 | 34000000 | 2954 | 0.98 |
| LoChr4.2 | LoChr4 | 23000000 | 25000000 | 2600 | 1.30 |
| LoChr5.1 | LoChr5 | 0 | 3000000 | 2978 | 0.99 |
| LoChr6.1 | LoChr6 | 2000000 | 5000000 | 2977 | 0.99 |
| LoChr6.2 | LoChr6 | 18000000 | 19000000 | 2500 | 2.50 |
| LoChr6.3 | LoChr6 | 28000000 | 29000000 | 2500 | 2.50 |
| LoChr8.2 | LoChr8 | 27000000 | 28000000 | 2500 | 2.50 |
| LoChr9.1 | LoChr9 | 0 | 1000000 | 2500 | 2.50 |
| LoChr9.2 | LoChr9 | 27000000 | 28000000 | 2500 | 2.50 |
| LoChr11.1 | LoChr11 | 0 | 1000000 | 2500 | 2.50 |
| LoChr13.1 | LoChr13 | 0 | 1000000 | 2500 | 2.50 |
| LoChr13.2 | LoChr13 | 9000000 | 10000000 | 2450 | 2.45 |
| LoChr14 | LoChr14 | 0 | 1000000 | 2400 | 2.40 |
| Total |  |  |  | 91550 |  |

^a^Chrmosomal naming referenced to the reference of genome

^b^average density = each number of oligos/ windows size

**Table S2. Synthetic details of the oligo library.**

| Probe | Primer-A^a^ | Primer-C^a^ | Primer-B^a^ | Probe color^b^ | |
| --- | --- | --- | --- | --- | --- |
| LoChr1.1 | PA15 | PA16 | PA2 | | Red |
| LoChr1.2 | PA15 | PA16 | PA2 | | Red |
| LoChr1.3 | PA15 | PA16 | PA2 | | Red |
| LoChr1.4 | PA15 | PA16 | PA2 | | Red |
| LoChr2.3 | PA15 | PA16 | PA4 | | Red |
| LoChr3.2 | PA15 | PA16 | D12 | | Red |
| LoChr4.1 | PA15 | PA16 | PA10 | | Red |
| LoChr4.3 | PA15 | PA16 | PA10 | | Red |
| LoChr5.2 | PA15 | PA16 | D1 | | Red |
| LoChr5.3 | PA15 | PA16 | D1 | | Red |
| LoChr7.1 | PA15 | PA16 | D5 | | Red |
| LoChr7.2 | PA15 | PA16 | D5 | | Red |
| LoChr7.3 | PA15 | PA16 | D5 | | Red |
| LoChr8.1 | PA15 | PA16 | D7 | | Red |
| LoChr10.1 | PA15 | PA16 | D24 | | Red |
| LoChr10.2 | PA15 | PA16 | D24 | | Red |
| LoChr12.1 | PA15 | PA16 | D28 | | Red |
| LoChr12.2 | PA15 | PA16 | D28 | | Red |
| LoChr11.2 | PA15 | PA16 | D27 | | Red |
| LoChr15 | PA15 | PA16 | D9 | | Red |
| LoChr2.1 | PA13 | PA14 | PA4 | | Green |
| LoChr2.2 | PA13 | PA14 | PA4 | | Green |
| LoChr3.1 | PA13 | PA14 | D12 | | Green |
| LoChr3.3 | PA13 | PA14 | D12 | | Green |
| LoChr4.2 | PA13 | PA14 | PA10 | | Green |
| LoChr5.1 | PA13 | PA14 | D1 | | Green |
| LoChr6.1 | PA13 | PA14 | D2 | | Green |
| LoChr6.2 | PA13 | PA14 | D2 | | Green |
| LoChr6.3 | PA13 | PA14 | D2 | | Green |
| LoChr8.2 | PA13 | PA14 | D7 | | Green |
| LoChr9.1 | PA13 | PA14 | D20 | | Green |
| LoChr9.2 | PA13 | PA14 | D20 | | Green |
| LoChr11.1 | PA13 | PA14 | D27 | | Green |
| LoChr13.1 | PA13 | PA14 | D29 | | Green |
| LoChr13.2 | PA13 | PA14 | D29 | | Green |
| LoChr14 | PA13 | PA14 | D14 | | Green |

^a^Primer-A, primer-B and primer-C represent the position of different specific primer in synthetic library system, and the position of three primers are shown in **Figure 1**.

^b^Probe color are selected type of fluorescence label in PCR amplification, including red is oligo carrying Cy3 direct label and green is oligo carrying FAM direct label.

**Table S3. Details of primer sequences.**

| Primer | Sequence (5’→3’) |
| --- | --- |
| PA1 | TCACCATCCACTCTAAACAC |
| PA3 | ACCCTCACCTATCAACTCAA |
| U12 | GGTCGTTTACTACTCATTCG |
| PA9 | ATCACACTCCAACTACAACC |
| U12 | CGTAGTAAGATATGTAGTCC |
| U2 | TGATATGCCGATTCCAAGCG |
| U5 | CTATACCACACATCTTAGTC |
| U7 | GTAGTACCTTCTGATAGTGA |
| U20 | GATTACTGCTACCATTGATC |
| U24 | GGCTGAGTTACACTACCTTA |
| U27 | ACAACACTCCTCGTTACACC |
| U28 | GCGAGTTCCTAGTGATACCG |
| U29 | CGGTAGGGATATTGTAACCA |
| U14 | GTTGAGTCACTAAGTTACTC |
| U9 | TTAGTCTGTGTAAATACCTC |
| PA2 | CACTTTACACCTCCACTCAT |
| PA4 | CTTCCGACCACTATACCTCT |
| D12 | TTATACATCATCGCAAACGG |
| PA10 | CCCTCACCTCTACACTAAAC |
| D12 | AACGATACGGTCTACGCCAC |
| D2 | AGTACCGACACACCGAAATC |
| D5 | ACAACCGCTCTTCGTTCACA |
| D7 | CTCGGGGTATGAGTAAAACC |
| D20 | ACTCTATCCAATCGTGTCCA |
| D24 | CCACTAGATAAGAGGTAACC |
| D27 | CGTCTGCTCTATATGACTGC |
| D28 | GTACACAAGACTGATAAGTC |
| D29 | GGATAGGTGCTACACTCTAC |
| D14 | TTGGAGGCACTCAATAAGTC |
| D9 | AGTGTGTACGGACTAATAAT |
| PA14 | CTTGCTGATATGGGACTTGA |
| PA16 | AGTAGGCAAGGTCAGATAAA |

**Table S4. The number of conserved sequences of the longan oligo-spanning regions among lychee and rambutan.**

| Probe Site | Lychee | Rambutan |
| --- | --- | --- |
| LoChr1.1 | 1330 | 914 |
| LoChr1.2 | 1049 | 622 |
| LoChr1.3 | 806 | 498 |
| LoChr1.4 | 1180 | 788 |
| LoChr2.1 | 1153 | 723 |
| LoChr2.2 | 1276 | 895 |
| LoChr2.3 | 955 | 709 |
| LoChr3.1 | 1096 | 792 |
| LoChr3.2 | 940 | 579 |
| LoChr3.3 | 853 | 563 |
| LoChr4.1 | 825 | 516 |
| LoChr4.2 | 912 | 514 |
| LoChr4.3 | 982 | 668 |
| LoChr5.1 | 715 | 468 |
| LoChr5.2 | 842 | 533 |
| LoChr5.3 | 1145 | 779 |
| LoChr6.1 | 542 | 379 |
| LoChr6.2 | 1093 | 679 |
| LoChr6.3 | 1248 | 934 |
| LoChr7.1 | 1361 | 918 |
| LoChr7.2 | 840 | 465 |
| LoChr7.3 | 1012 | 645 |
| LoChr8.1 | 1030 | 657 |
| LoChr8.2 | 965 | 654 |
| LoChr9.1 | 1224 | 847 |
| LoChr9.2 | 951 | 642 |
| LoChr10.1 | 415 | 286 |
| LoChr10.2 | 1228 | 784 |
| LoChr11.1 | 1265 | 918 |
| LoChr11.2 | 933 | 607 |
| LoChr12.1 | 883 | 592 |
| LoChr12.2 | 1272 | 882 |
| LoChr13.1 | 1188 | 821 |
| LoChr13.2 | 1125 | 726 |
| LoChr14 | 1346 | 938 |
| LoChr15 | 1231 | 901 |

**Table S5.** The longan special oligo-based barcode probe map on lychee and rambutan genomes.

| Probe | Lychee | | | | Rambutan | | | |
| --- | --- | --- | --- | --- | --- | --- | --- | --- |
|  | mapping number | | | Rate^c^ (%) | mapping number | | | Rate^c^ (%) |
|  | total | orthologous^a^ (%) | un-orthologous^b^ (%) |  | total | orthologous^a^ (%) | un-orthologous^b^ (%) |  |
| LoChr1 | 4365 | 4310 (98.74%) | 55 (1.26%) | 43.65 | 2822 | 2780 (98.51%) | 42 (1.49%) | 28.22 |
| LoChr2 | 3384 | 3354 (99.11%) | 30 (0.89%) | 45.61 | 2327 | 2284 (98.15%) | 43 (1.85%) | 31.36 |
| LoChr3 | 2889 | 1839 (63.66%) | 1050 (36.34%) | 36.32 | 1934 | 1827 (94.47%) | 107 (5.53%) | 24.31 |
| LoChr4 | 2719 | 2681 (98.60%) | 38 (1.40%) | 35.69 | 1698 | 1652 (97.29%) | 46 (2.71%) | 22.29 |
| LoChr5 | 2702 | 2643 (97.82%) | 59 (2.18%) | 33.87 | 1780 | 1720 (96.63%) | 60 (3.37%) | 22.31 |
| LoChr6 | 2883 | 2822 (97.88%) | 61 (2.12%) | 36.14 | 1992 | 1913 (96.03%) | 79 (3.97%) | 24.97 |
| LoChr7 | 3213 | 3171 (98.69%) | 42 (1.31%) | 42.84 | 2028 | 1996 (98.42%) | 32 (1.58%) | 27.04 |
| LoChr8 | 1995 | 1950 (97.74%) | 45 (2.26%) | 39.9 | 1311 | 1243 (94.81%) | 68 (5.19%) | 26.22 |
| LoChr9 | 2175 | 1933 (88.87%) | 242 (11.13%) | 43.5 | 1489 | 1440 (96.71%) | 49 (3.29%) | 29.78 |
| LoChr10 | 1643 | 1596 (97.14%) | 47 (2.86%) | 30.69 | 1070 | 982 (91.78%) | 88 (8.22%) | 19.99 |
| LoChr11 | 2198 | 2126 (96.72%) | 72 (3.28%) | 43.96 | 1525 | 1369 (89.77%) | 156 (10.23%) | 30.5 |
| LoChr12 | 2155 | 2118 (98.28%) | 37 (1.72%) | 43.98 | 1474 | 1423 (96.54%) | 51 (3.46%) | 30.08 |
| LoChr13 | 2313 | 2278 (98.49%) | 35 (1.51%) | 46.73 | 1547 | 1530 (98.90%) | 17 (1.10%) | 31.25 |
| LoChr14 | 1346 | 1323 (98.29%) | 23 (1.71%) | 56.08 | 938 | 911 (97.12%) | 27 (2.88%) | 39.08 |
| LoChr15 | 1231 | 1225 (99.51%) | 6 (0.49%) | 49.24 | 901 | 885 (98.22%) | 16 (1.78%) | 36.04 |

^a^The rate of orthologous: the number of orthologous sequences/the total of mapping sequences.

^b^The rate of un-orthologous: the number of un-orthologous sequences/the total of mapping sequences.

^c^Rate: the total of mapping sequences/the total of special oligo barcode sequences.
